# An analysis of lithium requesting across three hospital trusts in the UK: many people are managed with lithium levels below the current nice guidance lower limit

**DOI:** 10.1192/bjo.2021.131

**Published:** 2021-06-18

**Authors:** Adrian Heald, Ceri Parfitt, Chris Duff, Jonathan Scargill, Lewis Green, Anthony Fryer

**Affiliations:** 1 University of Manchester; 2 Royal Stoke University Hospital; 3 Royal Stoke Hospital; 4 North Manchester General Hospital; 5 St Helens and Knowsley Hospital

## Abstract

**Aims:**

This study examined lithium results and requesting patterns over a 6-year period, and compared these to guidance.

**Background:**

Bipolar disorder is the 4th most common mental health condition, affecting ~1% of UK adults. Lithium is an effective treatment for prevention of relapse and hospital admission, and is recommended by NICE as a first-line treatment.

We have previously shown in other areas that laboratory testing patterns are highly variable with sub-optimal conformity to guidance.

**Method:**

Lithium requests received by Clinical Biochemistry Departments at the University Hospitals of North Midlands, Salford Royal Foundation Trust and Pennine Acute Hospitals from 2012–2018 were extracted from Laboratory Information and Management Systems (46,555 requests; 3,371 individuals). We categorised by request source, lithium concentration and re-test intervals.

**Result:**

Many lithium results were outside the NICE therapeutic window (0.6–0.99mmol/L); 49.3% were below the window and 6.1% were above the window (median [Li]:0.61mmol/L). A small percentage were found at the extremes (3.2% at <0.1mmol/L, 1.0% at >1.4mmol/L). Findings were comparable across all sites.

For requesting interval, there was a distinct peak at 12 weeks, consistent with guidance for those stabilised on lithium therapy. There was no peak evident at 6 months, as recommended for those <65 years old on unchanging therapy. There was a peak at 0–7 days, reflecting those requiring closer monitoring (e.g. treatment initiation or results suggesting toxicity).

However, 77.6% of tests were requested outside expected testing frequencies.

**Conclusion:**

We showed: (a) lithium levels are often maintained at the lower end of the NICE recommended therapeutic range (and the BNF range: 0.4-1.0mmol/L); (b) patterns of lithium results and testing frequency are comparable across three sites with differing models of care; (c) re-test intervals demonstrate a noticeable peak at the recommended 3-monthly interval, but not at 6-monthly intervals; (d) Many tests were repeated outside these expected frequencies (contrary to NICE guidance).

